# Cost of childhood acute otitis media in primary care in the Netherlands: economic analysis alongside a cluster randomised controlled trial

**DOI:** 10.1186/s12913-021-06157-1

**Published:** 2021-03-04

**Authors:** Rick T. van Uum, Roderick P. Venekamp, Clémence T. B. Pasmans, G. Ardine de Wit, Alies Sjoukes, Alma C. van der Pol, Roger A. M. J. Damoiseaux, Anne G. M. Schilder

**Affiliations:** 1 Julius Center for Health Sciences and Primary Care, University Medical Center Utrecht, University Utrecht, P.O. Box 85500, 3508 GA Utrecht, The Netherlands; 2 Centre for Nutrition, Prevention and Healthcare, National Institute of Public Health and the Environment, Bilthoven, The Netherlands; 3 evidENT, Ear Institute, University College London, London, UK; 4 National Institute for Health Research, University College London Hospitals Biomedical Research Centre, London, UK

**Keywords:** Acute otitis media, Pain management, Healthcare resources use, Societal cost of AOM

## Abstract

**Background:**

Acute otitis media (AOM) is among the most common paediatric conditions managed in primary care. Most recent estimates of the cost of AOM date from a decade ago and lack a full societal perspective. We therefore explored the societal cost of childhood AOM in the Netherlands within the setting of a trial comparing the effectiveness of an intervention aimed at educating general practitioners (GPs) about pain management in AOM compared to usual care.

**Methods:**

Economic analysis alongside a cluster randomised controlled trial conducted between February 2015 and May 2018 in 37 practices (94 GPs). In total, 224 children with AOM were included of which 223 (99%) completed the trial (intervention: *n* = 94; control: *n* = 129). The cost of AOM due to health care costs, patient and family costs, and productivity losses by parent caregivers were retrieved from study diaries and primary care electronic health records, during 28-day follow-up. We calculated mean cost (€ and $) per AOM episode per patient with standard deviations (SD, in €) regardless of study group assignment because there was no clinical effect of the trial intervention. In sensitivity analysis, we calculated cost in the intervention and usual care group, after exclusion of extreme outliers.

**Results:**

Mean total AOM cost per patient were €565.93 or $638.78 (SD €1071.01); nearly 90% of these costs were due to productivity losses experienced by parents. After exclusion of outliers, AOM cost was €526.70 or $594.50 (SD €987.96) and similar in the intervention and usual care groups: €516.10 or $582.53 (SD €949.69) and €534.55 or $603.36 (SD €920.55) respectively.

**Conclusions:**

At €566 or $639 per episode, societal cost of AOM is higher than previously known and mainly driven by productivity losses by children’s parents. Considering its high incidence, AOM poses a significant economic burden that extends beyond direct medical costs.

**Trial registration:**

Netherlands Trial Register no. NTR4920: http://www.trialregister.nl/trialreg/admin/rctview.asp?TC=4920.

**Supplementary Information:**

The online version contains supplementary material available at 10.1186/s12913-021-06157-1.

## What’s known on this subject

Most recent estimates of the cost of acute otitis media (AOM), a common paediatric condition, date from a decade ago and lack a full societal perspective.

## What this study adds

The societal cost of AOM in the Netherlands is €566 or $639 per episode, which is more than previously estimated. Ninety percent of costs are related to productivity losses by children’s parents.

## Background

With a global incidence of 10.8 episodes per 100 children each year [1, 2], acute otitis media (AOM) is among the most common paediatric conditions and reasons for doctors’ visits, antibiotic prescribing and surgery in young children [3]. AOM is associated with considerable resource use, in healthcare and beyond [4–6].

Previous estimates of the cost of AOM do not provide the full picture [6, 7]. In 2017, the cost of an AOM episode in the United States (US) was estimated at $314 (€278; currency conversion as of July 6th, 2020), but this estimate included health care resources use only [7]. A decade ago, the cost of AOM in the Netherlands and the UK was estimated at €332 and €752, respectively [6]. These figures included health care costs and patient and family costs, but not costs of productivity losses of both parents.

More important, AOM guidelines promoting more accurate diagnosis and judicious use of antibiotics, and pneumococcal conjugate vaccination, have been introduced in recent years, which may have changed the burden of AOM in terms of incidence, clinical picture and cost [8].

We therefore set out to gather robust and up-to-date estimates of the cost of AOM from a societal perspective, within the setting of a cluster randomised controlled trial of an intervention aimed at educating general practitioners (GPs) about pain management in children with AOM [9].

## Methods

### Design and participants

The design of the cluster randomised controlled trial and results focused on clinical effectiveness of the intervention have been reported in detail elsewhere [9, 10]. In short, 37 GP practices were randomly assigned, using a computerised minimisation strategy, to either the intervention or the control group.GPs in practices allocated to the intervention group were offered a blended educational program (online and face-to-face training); they were trained to discuss pain management with parents using an information leaflet, and prompted to prescribe analgesics (paracetamol, and ibuprofen as add-on in case of insufficient pain relief) in weight-appropriate dosage. GPs in the practices allocated to the control group provided usual care. Management decisions, including antibiotic prescribing, were at the discretion of the GP. Children aged 6 months to 10 years with a GP-confirmed diagnosis of AOM (according to Dutch guidelines [11]) were eligible for participation, and were recruited by their GP. After inclusion, participants were followed for 28 days to capture the full range of the AOM episode, including all associated costs. The trial’s primary outcome was parent-reported mean ear pain score (scale 0–10) over the first three days;

### Data collection

Data on health care costs, including GP consultations, prescription medication, specialist referrals, and hospital admissions, during the 28-day follow-up period were extracted from children’s primary care electronic health records. Data on patient and family costs due to the AOM episode were retrieved from diaries completed daily by the parents for the duration of follow-up. These diaries included questions on travel expenses, costs of over-the-counter (OTC) medication, and costs of childcare, related to the AOM episode. Data on productivity losses by parents of the children were retrieved from a questionnaire (iMTA Productivity Cost Questionnaire (iPCQ) [12] completed at day 28.

### Resource use and valuation

Economic analyses were conducted using a societal perspective; including health care costs, patient and family costs, and costs of parental productivity losses. A detailed overview of unit costs for all cost items included in the study is shown in Table 1 and Supplementary Table 1.

**TABLE 1:** UNIT COSTS

Resources	Unit	Cost estimate		Source
		€	$^§^	
**Health care costs**				
Direct costs				
GP consultation	consultation	33.76	38.11	ZIN guideline
GP home visit	consultation	51.16	57.75	ZIN guideline
GP telephone	consultation	17.39	19.63	ZIN guideline
OPD visit	visit	81.85	92.39	ZIN guideline
ED visit	visit	264.99	299.10	ZIN guideline
Admission	day	453.25	511.59	ZIN guideline
Pharmacist fee	prescription	12.28	13.86	ZIN guideline
Prescription medication				
*Antibiotics*				
*Amoxicillin*	prescription^†^	3.43	3.87	www.medicijnkosten.nl
*Amoxicillin-clavulanate*	prescription^†^	3.43	3.87	www.medicijnkosten.nl
*Azitromycin*	prescription^†^	3.97	4.48	www.medicijnkosten.nl
*Cotrimoxazole*	prescription^†^	2.81	3.17	www.medicijnkosten.nl
*Clarithromycin*	prescription^†^	9.51	10.73	www.medicijnkosten.nl
*Ear drops*				
*Otalgan®*	bottle	10.99	12.40	www.medicijnkosten.nl
*Sofradex®*	bottle	10.56	11.92	www.medicijnkosten.nl
*Ofloxacin*	bottle	0.50	0.56	www.medicijnkosten.nl
*Bacicoline B drops*	bottle	14.70	16.59	www.medicijnkosten.nl
Intervention costs^**‡**^	patient	50.85	57.40	collective labour agreement for GPs
**Patient and family costs**				
Travel expenses				
*Fuel costs*	kilometer	0.19	0.21	ZIN guideline
*Parking costs*	visit	3.07	3.47	ZIN guideline
Over-the-counter medication				
*Paracetamol*	1000 mg	1.41	1.59	www.medicijnkosten.nl
*Ibuprofen*	1000 mg	2.50	2.82	www.medicijnkosten.nl
*Otalgan®*	bottle	10.99	12.40	www.medicijnkosten.nl
*Xylometazoline nasal spray*	bottle	2.08	2.35	retail prices**
*Otrivin® nasal spray*	bottle	4.59	5.18	retail prices**
*Sodium chloride nasal spray*	bottle	2.95	3.33	retail prices**
Complementary medicine*	bottle	4.95–14.35	5.59–16.20	retail prices**
*Cough syrup*	bottle	10.95	12.36	retail prices**
Childcare costs	one hour	14.32	16.16	ZIN guideline
**Productivity loss**				
Productivity costs father	one hour	38.78	43.77	iPCQ questionnaire
Productivity costs mother	one hour	32.33	36.49	iPCQ questionnaire
Unpaid work	one hour	14.32	16.16	iPCQ questionnaire

ED: emergency department; GP: general practitioner, iPCQ: iMTA Productivity Cost Questionnaire, mg: milligrams, OPD: out-patient department; ZIN: Zorginstituut Nederland (Netherlands Health Institute)

^§^ currency conversion as of July 6th, 2020 (1 euro = 1.12872 USD, www.xe.com/ucc)

^†^ one or two units per patient, depending on weight

^**‡**^ calculated as one-hour GP wage costs (time spent on the training) times the number of GPs in intervention group times printing costs, divided the number of patients included in the intervention group

* full display of prices of specific complementary medicine products is available online in Supplement Table 1

** prices are based on retail prices of the most used retailers in the Netherlands: Albert Heijn, Etos, Kruidvat and DA. Available from www.ah.nl, www.kruidvat.nl and www.da.nl

Costs of prescription medication were estimated by using a publicly available Dutch database of current drug prices [13], increased with a pharmacist’s charge. Costs of GP consultation, as well as hospital outpatient department (OPD) visits, emergency department (ED) visits and hospital admissions were based on Dutch guidelines for pharmacoeconomic evaluation [14]. These guidelines include reference cost figures for the use in health economic evaluations for common types of healthcare use. Using a consumer price index (CPI) [15], 2014 costs from these guidelines were corrected for inflation up to 2017, the base year for cost calculations.

Costs of OTC and complementary medication were based on average retail prices used in the Netherlands [16–18]. Childcare costs were used as reported by parents in the diary. Travel costs were based on Dutch guidelines for pharmacoeconomic evaluation, and corrected for inflation up to 2017 [14, 15].

For productivity losses, we calculated costs of absenteeism (being absent from work), presenteeism (being less productive while at work) and not being able to do unpaid work. Accumulating these three subtypes of productivity costs, we calculated a composite total productivity loss per parent caregiver.

### Analysis

In the current analyses, we deviated from our initial research protocol [9] in two ways. First, considering the absence of clinical effectiveness of the intervention at trial [10], we primarily combined data from the intervention and usual care group to estimate AOM cost in the overall trial population. Second, we refrained from calculating incremental cost-effectiveness ratios (ICER) for the same reason.

Given the short-term duration of the trial, neither costs nor benefits were discounted. We imputed ten times for relevant missing data using the SPSS multiple imputation function [19], and subsequently pooled results using Rubin’s rule [20].

In primary analysis, we calculated mean costs per patient with standard deviations (SD) regardless of study group assignment. In sensitivity analysis, we compared costs in the intervention and usual care group, after exclusion of extreme outliers and the intervention costs, to define whether there were significant differences between the intervention and usual care group.

All analyses were performed in SPSS version 25.0 (SPSS Inc., IBM Corporation, Chicago, IL).

## Results

Between February 2015 and May 2018, 94 GPs in 37 GP practices across the Netherlands recruited 224 children (intervention *n* = 94; control *n* = 130) children aged 6 months to 10 years diagnosed with AOM and ear pain to the trial.

Table 2 shows the baseline characteristics of participating children. The baseline characteristics of GP practices (i.e. number of patients, % of patients < 10 years, setting), individual GPs (i.e. age, experience), as well as children were generally well-balanced. Participants had a median age of 40 months (IQR 16–64 months, full range 6 months to 9 years and 10 months), 53.8% were boys. 64.6% had unilateral AOM, and 15.7% had AOM (unilateral or bilateral) with otorrhea. Most patients had had ear pain prior to consulting their GP (86.9%) for a median of 2 days (IQR 0.5–3.5); fewer patients had had fever prior to consulting (64.3%, median number of days 2, IQR 0.5–3.5).

**TABLE 2:** BASELINE CHARACTERISTICS

	Total group *n* = 223; 37 practices	Intervention *n* = 94; 19 practices	Usual care *n* = 129; 18 practices
**Characteristic**			
**Age (months)**^**†**^	40 (16–64)	38 (13–62)	43 (20–66)
**Sex (boys)**	120 (53.8)	54 (57.4)	66 (51.2)
**Medical history**			
Recurrent AOM	26 (11.7)	14 (14.9)	12 (9.3)
Recurrent URTI	24 (10.8)	13 (13.8)	11 (8.5)
Previous ENT surgery	23 (10.3)	15 (16.0)	8 (6.2)
Atopic constitution	24 (10.8)	16 (17.0)	8 (6.2)
**Symptoms prior to consultation (parent-reported)**			
Ear pain (yes/no)*	166 (86.9)	75 (90.4)	91 (84.3)
*Number of days*^†^	2 (0.5–3.5)	2 (0.5–3.5)	3 (1–5)
Otorrhoea*	28 (15.7)	12 (15.4)	16 (16.0)
*Number of days*^†^	*0 (0–0)*	*2 (0–5)*	*2 (0–4.5)*
Fever*	126 (64.3)	50 (60.2)	76 (67.3)
*Number of days*^†^	*2 (0.5–3.5)*	*2 (1–3)*	*3 (2–4)*
**Physical examination**			
Temperature in °C^‡^	37.8 ± 1.0	37.6 ± 0.9	37.9 ± 1.1
Ill appearance	43 (20.0)	18 (19.6)	25 (20.3)
Unilateral AOM	144 (64.6)	65 (69.1)	79 (61.2)
*Redness*	*132 (59.2)*	*60 (92.3)*	*72 (91.1)*
*Bulging*	*81 (36.3)*	*29 (44.6)*	*52 (65.8)*
*Otorrhoea*	*12 (5.4)*	*4 (6.2)*	*8 (10.1)*
Bilateral AOM	79 (35.4)	29 (30.9)	50 (38.8)
*Redness*	*74 (33.2)*	*29 (100)*	*45 (90.0)*
*Bulging*	*53 (23.8)*	*17 (58.6)*	*36 (72.0)*
*Otorrhoea*	*8 (3.6)*	*3 (10.3)*	*5 (10.0)*
**Symptoms at baseline (parent-reported)**			
Proportion of children with ear pain*	204 (98.1)	87 (98.9)	117 (97.5)
Proportion of children with fever*	107 (54.9)	40 (48.2)	67 (59.8)
Antibiotic prescriptions**	92 (41.3)	36 (38.3)	56 (43.4)

Values are numbers (percentages) unless stated otherwise

°C: degrees Celsius; AOM: acute otitis media; ENT: ear, nose, throat; GP: general practitioner; URTI: upper respiratory tract infection

^†^ median with IQR, ^‡^ mean with SD

* missings: otorrhea prior to consultation (45), ear pain prior to consultation (32), fever prior to consultation (21), temperature (10), ill appearance (8), ear pain at baseline (15), fever at baseline (22)

** number (and percentage) of children prescribed one (or more) antibiotics

Data on health care costs were available for 223 children (99.6%). Available data on patient and family costs varied per subcategory: we had data on travel expenses for 223 children (99.5%), on childcare costs for 162 children (72.3%) and on OTC medication for 206 children (92.0%). Data on productivity losses were available for 181 children (80.8%). Some parents did not complete all questionnaires, hence the variability in missing data. Missing data appeared to be randomly divided, we had no indication for selective missing data.

Mean total cost of AOM per patient was €565.93 or $638.78 (SD €1071.01), with high interindividual variation. Currency conversion as of July 6th, 2020 (1 euro = 1.12872 USD, www.xe.com/ucc). Full details on costs in each category are shown in Table 3.

**TABLE 3:** USE OF RESOURCES AND MEAN COSTS (IN €) PER CHILD

Resources	Mean costs			
	no. used (n, %)	costs, in € (mean ± SD)	costs, in $ (mean ± SD)	
GP consultation, initial visit	224 (100.0)	33.76 ± 0.00	38.11 ± 0.00	
GP consultation, follow-up visit	74 (33.1)	16.04 ± 1.77	18.10 ± 2.00	
GP home visit	0 (0)	0.00 ± 0.00	0.00 ± 0.00	
GP telephone	40 (17.9)	3.51 ± 0.53	3.96 ± 0.60	
OPD visit	7 (3.1)	3.30 ± 1.41	3.72 ± 1.59	
ED visit	1 (0.0)	1.19 ± 1.19	1.34 ± 1.34	
Admission	1 (0.0)	10.16 ± 10.16	11.47 ± 11.47	
Pharmacist fee	102 (45.7)	6.55 ± 0.53	7.39 ± 0.60	
Prescription medication				
*Antibiotics*				
*Amoxicillin*	80 (35.9)	1.86 ± 0.19	2.10 ± 0.21	
*Amoxicillin-clavulanate*	6 (2.7)	0.14 ± 0.06	0.16 ± 0.07	
*Azitromycin*	6 (2.7)	0.14 ± 0.06	0.16 ± 0.07	
*Cotrimoxazole*	2 (0.01)	0.03 ± 0.02	0.03 ± 0.02	
*Clarithromycin*	1 (0.0)	0.04 ± 0.04	0.05 ± 0.05	
*Ear drops*				
*Otalgan®*	10 (4.5)	0.49 ± 0.15	0.55 ± 0.17	
*Sofradex®*	3 (1.3)	0.24 ± 0.14	0.27 ± 0.16	
*Ofloxacin*	1 (0.0)	0.00 ± 0.00	0.00 ± 0.00	
*Bacicoline B drops*	2 (0.01)	0.13 ± 0.09	0.15 ± 0.10	
**Total healthcare costs**		**77.60 ± 160.89**	**87.59 ± 181.60**	
**Patient and family costs**				
Travel expenses				
*Fuel costs*	9 (4.0)	0.09 ± 0.04	0.10 ± 0.05	
*Parking costs*	9 (4.0)	0.21 ± 0.09	0.24 ± 0.10	
Over-the-counter medication				
*Paracetamol*	191 (85.7)	2.55 ± 3.10	2.88 ± 3.50	*
*Ibuprofen*	79 (35.4)	0.62 ± 1.34	0.70 ± 1.51	*
*Xylometazoline nasal spray*	29 (13.0)	0.87 ± 2.77	0.98 ± 3.13	*
*Otrivin® nasal spray*	17 (7.6)	0.35 ± 1.22	0.40 ± 1.38	*
*Sodium chloride nasal spray*	44 (19.7)	0.62 ± 1.30	0.70 ± 1.47	*
*Complementary medicine*	33 (14.8)	3.38 ± 12.28	3.82 ± 13.86	*
*Cough syrup*	9 (4.0)	1.21 ± 7.97	1.37 ± 9.00	*
Childcare costs	27 (12.1)	3.17 ± 18.45	3.58 ± 20.82	*
**Total patient and family costs**		**13.07 ± 23.58**	**14.75 ± 26.62**	
**Productivity loss**				
Father	79 (35.4)			
*Absenteeism*		106.13 ± 354.05	119.79 ± 399.62	*
*Presenteeism*		41.90 ± 180.97	47.29 ± 204.26	*
*Unpaid work*		38.06 ± 173.75	42.96 ± 196.12	*
*Total*		186.09 ± 529.31	210.04 ± 597.44	*
Mother	126 (56.5)			
*Absenteeism*		139.74 ± 637.66	157.73 ± 719.74	*
*Presenteeism*		52.50 ± 217.53	59.26 ± 245.53	*
*Unpaid work*		96.92 ± 256.35	109.40 ± 289.35	*
*Total*		289.16 ± 786.73	326.38 ± 888.00	*
**Total productivity losses**^**‡**^		**475.26 ± 1045.94**	**536.44 ± 1180.57**	
**Total**				
**Total healthcare costs**		**77.60 ± 160.89**	**87.59 ± 181.60**	
**Total patient costs**		**13.07 ± 23.58**	**14.75 ± 26.62**	
**Total productivity losses**		**475.26 ± 1045.94**	**536.44 ± 1180.57**	
**Total costs**		**565.93 ± 1071.01**	**638.78 ± 1208.87**	

ED: emergency department; GP: general practitioner, OPD: out-patient department, SD: standard deviation

^†^ one admission for mastoiditis (duration: 5 days)

^**‡**^ Per child, calculated as productivity losses of mother and/or father combined

*missing values were imputed

Mean total health care costs were €77.60 or $87.59 (SD €160.89). The largest contributors to these costs were GP consultations and hospital admissions, at €49.80 or $56.21 (SD €1.77; 64.2%) and €10.16 or $11.47 (SD €10.16; 13.1%) per patient, respectively. Prescription medication costs attributed for €3.07 or $3.46 (SD €0.18; 3.9%).

Families spent on average €13.07 of $14.75 (SD €23.58) out of their own pocket. Main contributors were analgesics use, complementary medicine and childcare costs, at €3.17 or $3.58 (SD €2.59; 24.3%), €3.38 or $3.81 (SD €12.28; 25.8%) and €3.17 or $3.58 (SD €18.45; 24.3%) per patient, respectively.

Parental productivity losses contributed the largest share of costs, adding up to €475.26 or $536.42 (SD €1045.95) per patient. Overall, 56% of mothers reported productivity losses, compared to 36% of fathers. In mothers, costs were primarily related to absenteeism and unpaid work; for fathers, absenteeism was the major contributor. Productivity losses for mothers and fathers was €289.16 or $326.37 (SD €60.8; 25.8% of total cost) and €186.09 or $210.04 (SD €529.31; 39.2% of total cost) per patient, respectively.

Extreme outliers were identified in two children in the usual care group: one five-day hospitalisation for acute mastoiditis and extreme productivity losses reported by parents in another child. To study how these outliers influenced the average cost estimate, we performed an additional analysis, from which these two subject were excluded. When excluding these children from analysis, overall mean total cost per patient was slightly lower, but still had high interindividual variation: €526.70 or $ 594.50 (SD €987.96). Cost was comparable between intervention and usual care group, at €516.10 or $582.53 (SD €949.69) and €534.55 or $603.36 (SD €920.55) per patient, respectively. Supplementary Table 2 displays a comprehensive overview of costs in the separate groups.

## Discussion

The cost of an AOM episode to the Dutch society were found to be €566 ($639), of which 90% is due to productivity losses by parents. Each year, GPs diagnose 110,000 AOM episodes in children under the age of ten [1], which brings the total cost of AOM in the Netherlands to €62.3 million annually. The true economic burden of AOM is probably higher with one in two episodes with AOM symptoms self-managed by parent caregivers [23], although it is unclear how high productivity losses are in parents self-managing AOM episodes (presumably lower than in those consulting their GP).

Our cost estimate of €566 per AOM episode is higher than the €332 (2020: €270 after adjustment for inflation and purchasing power) [15, 24] that was described about a decade ago [6]. This difference may be related to our more accurate method of cost data collection: we collected detailed cost data prospectively over 28 days post AOM diagnosis by a parent diary and questionnaire whereas Wolleswinkel et al [6] gathered retrospective estimates from parents participating in a consumer panel. Furthermore, this difference may be explained by a change in incidence and burden of disease, as recent years saw guidelines [11, 25, 26] introduced that promote more accurate diagnosis and judicious use of antibiotics as well as the introduction of the pneumococcal conjugate vaccination. This may have resulted in a higher proportion of more severe AOM cases that are presented to clinicians, with milder cases self-managed by parents at home.

From an international perspective, our cost estimate of €566 is lower than one found in the UK (€752, 2020: €910 after adjustment) [6, 15, 24]. Apart from differences in cost data collection, UK costs are higher due to higher costs of medical facilities, and due to a larger proportion of children experiencing symptoms of AOM presenting to emergency hospital services as well as higher antibiotic prescription rates [6, 27]. United States data available so far include only cost of health care resource use for AOM and in light of our results represent an underestimate of the true cost of AOM to societies [4].

Our cost estimates show a high interindividual variation, both in the primary analysis, as well as in the sensitivity analysis in which we exclude two outliers with considerable resource use. This interindividual variation was largely a consequence of a variation in parental productivity loss (see Table 3), with some parents reporting very little absence (or reduced productivity at work), but others considerable productivity loss.

### Strengths and limitations

This economic analysis provides a detailed and up-to-date account of the cost of childhood AOM in the Netherlands from a societal perspective; we prospectively collected detailed cost data using a daily symptom diary including a productivity loss questionnaire, and review of medical records. Although this cost study was embedded in a trial, we are confident that our estimates reflect those experienced in day-to-day practice in the Netherlands because [1] our pragmatic RCT left most treatment decisions at the GP’s discretion and [2] the clinical course of AOM in children in our study match previous studies [21, 28]. The intervention at trial had no effect on clinical outcomes and AOM cost were similar in the intervention and control group. The antibiotic prescription rate was lower in our trial context than in standard Dutch practice (41.3% vs. 55.0%), but similar in both treatment groups. Hence, the presented cost figures might be slightly lower than in daily Dutch practice, although the cost of prescription medication is minimal compared to overall cost.

This study has some limitations. AOM cost estimates are a reflection of countries’ health care systems and practices. Dutch GPs act as ‘gatekeepers’ to the healthcare system both in and out of office-hours; they manage all cases of AOM initially, and only refer to secondary care in case of complications. This is different from many other countries, where for example AOM is managed predominantly by community paediatricians or emergency hospital services [6, 7]. Importantly, for decades, Dutch GPs have practiced a watchful waiting strategy for AOM, resulting in half the antibiotic prescriptions compared to the UK (72.5 vs. 164 per 1000 child years) [22, 27]. In the US, 86% of doctor consultations for AOM ends with an antibiotic prescription [29]. Concerning study methods, some cost data were missing at-random in our study (10% for OTC medication, 19.2% for productivity loss, and 27.7% for childcare costs). We used multiple imputation techniques to handle these missing data, and minimise the impact on our analysis [20]. Furthermore, data on symptoms and resource use during follow-up were captured from parent-reported surveys in a patient diary, which parents filled in every day. Resources use (i.e. antibiotic and medication use, as well as GP and hospital visits) were cross-checked by collecting these from the patients’ medical files. As such, we have aimed to minimise the risk of recall bias. We captured parental productivity loss through a questionnaire that parents filled in at the end of the 28-day follow-up, risk of recall bias cannot be excluded for this questionnaire.

## Conclusions

At €566 per episode, and an estimated €62 million annually, societal cost of AOM is higher than previously known and mainly driven by productivity losses by children’s caregivers. Considering its high incidence, AOM poses a significant economic burden to society that extends beyond medical costs, close to €62 million annually in the Netherlands alone.

## Supplementary Information

**Additional file 1: Supplementary Table 1.** Complementary medicine unit costs.

**Additional file 2: Supplementary Table 2.** Sensitivity analysis 1. Use of resources and mean costs (in €) per child (imputed, two outliers excluded).

## Data Availability

Data generated during the study will be made available from the corresponding author on reasonable request.

## Abbreviations

AOM: Acute otitis media
CPI: Consumer price index
ED: Emergency department
ENT: Ear, nose, throat
GP: General Practitioner
ICER: Incremental cost-effectiveness ratio
ILI: Influenza-like illness
iPCQ: iMTA Productivity Cost Questionnaire
OPD: Out-patient department
PIM-POM: Pain Intensity Monitoring in Paediatric Otitis Media SD Standard deviation
UK: United Kingdom
US: United States
