# Supplementary material for: Cost of childhood acute otitis media in primary care in the Netherlands: economic analysis alongside a cluster randomised controlled trial
Source: BMC Health Serv Res. 2021 Mar 4;21:193. doi: 10.1186/s12913-021-06157-1 (PMC7931516; doi:10.1186/s12913-021-06157-1)
Supplement: Supplementary file 1 — Additional file 1: Supplementary Table 1. Complementary medicine unit costs. [file 12913_2021_6157_MOESM1_ESM.docx]

**SUPPLEMENTARY TABLE 1. COMPLEMENTARY MEDICINE UNIT COSTS**

| **Resources** | **Unit** | **Cost estimate** | | | **Source** |
| --- | --- | --- | --- | --- | --- |
|  |  | **€** | **$** |  |  |
| VSM Plantago Major | bottle | 12.62 | 14.24 |  | retail prices* |
| Echinaforce | bottle | 8.84 | 9.98 |  | retail prices* |
| Chamodent | bottle | 6.67 | 7.53 |  | retail prices* |
| VSM Rinikind | bottle | 4.95 | 5.59 |  | retail prices* |
| Oteel | bottle | 14.35 | 16.20 |  | retail prices* |
| Luuf | tube | 6.24 | 7.04 |  | retail prices* |
| VSM Tussikind | bottle | 6.44 | 7.27 |  | retail prices* |
| VSM Cinuforce | bottle | 6.85 | 7.73 |  | retail prices* |
| VSM airway drops | bottle | 10.59 | 11.95 |  | retail prices* |
| Juglagina | bottle | 10.45 | 11.80 |  | retail prices* |

*prices are based on retail prices of the most used retailers: Albert Heijn, Etos, Kruidvat and DA. Available from [www.ah.nl](http://www.ah.nl), [www.kruidvat.nl](http://www.kruidvat.nl) and [www.da.nl](http://www.da.nl)
