# Supplementary material for: Cost of childhood acute otitis media in primary care in the Netherlands: economic analysis alongside a cluster randomised controlled trial
Source: BMC Health Serv Res. 2021 Mar 4;21:193. doi: 10.1186/s12913-021-06157-1 (PMC7931516; doi:10.1186/s12913-021-06157-1)
Supplement: Supplementary file 2 — Additional file 2: Supplementary Table 2. Sensitivity analysis 1. Use of resources and mean costs (in €) per child (imputed, two outliers excluded). [file 12913_2021_6157_MOESM2_ESM.docx]

**SUPPLEMENTARY TABLE 2. SENSITIVITY ANALYSIS 1**

**USE OF RESOURCES AND MEAN COSTS (IN €) PER CHILD (IMPUTED, TWO OUTLIERS EXCLUDED)**

| **Resources** | **Treatment** | | | | | | | | |  |
| --- | --- | --- | --- | --- | --- | --- | --- | --- | --- | --- |
|  | **Intervention (n=94)** | | | | **Control (n=127)** | | | | |  |
|  |  | **No. used**  **(n, %)** | **costs, in €**  **(mean ± SD)** | **costs, in $**  **(mean ± SD)** | |  | **No. used**  **(n, %)** | **costs, in €**  **(mean ± SD)** | **costs, in $**  **(mean ± SD)** | |
| **Health care costs** | | | | | | | | | |  |
| GP consultation, initial visit |  | 94 (100) | 33.76 ± 0.00 | 38.11 ± 0.00 | |  | 127 (100) | 33.76 ± 0.00 | 38.11 ± 0.00 | |
| GP consultation, follow-up visit |  | 40 (42.6) | 21.19 ± 29.70 | 23.92 ± 33.52 | |  | 32 (25.2) | 11.70 ± 22.84 | 13.21 ± 25.78 | |
| GP home visit |  | 0 (0.0) | 0.00 ± 0.00 | 0.00 ± 0.00 | |  | 0 (0.0) | 0.00 ± 0.00 | 0.00 ± 0.00 | |
| GP telephone |  | 17 (18.1) | 3.52 ± 7.89 | 3.97 ± 8.91 | |  | 23 (18.1) | 3.56 ± 8.06 | 4.02 ± 9.10 | |
| OPD visit |  | 6 (6.4) | 6.97 ± 30.98 | 7.87 ± 34.97 | |  | 1 (0.8) | 0.64 ± 7.32 | 0.72 ± 8.26 | |
| ED visit |  | 1 (1.1) | 2.82 ± 27.33 | 3.18 ± 30.85 | |  | 0 (0.0) | 0.00 ± 0.00 | 0.00 ± 0.00 | |
| Admission |  | 0 (0.0) | 0.00 ± 0.00 | 0.00 ± 0.00 | |  | 0 (0.0) | 0.00 ± 0.00 | 0.00 ± 0.00 | |
| Pharmacist fee |  | 67 (71.3) | 10.95 ± 10.35 | 12.36 ± 11.68 | |  | 76 (59.8) | 8.48 ± 9.16 | 9.57 ± 10.34 | |
| Prescription medication |  |  |  |  | |  |  |  |  | |
| *Antibiotics* |  |  |  |  | |  |  |  |  | |
| *Amoxicillin* |  | 26 (27.7) | 1.43 ± 2.52 | 1.61 ± 2.84 | |  | 53 (41.7) | 2.18 ± 3.00 | 2.46 ± 3.39 | |
| *Amoxicillin-clavulanate* |  | 3 (3.2) | 0.18 ± 1.05 | 0.20 ± 1.19 | |  | 3 (2.4) | 0.11 ± 0.75 | 0.12 ± 0.85 | |
| *Azitromycin* |  | 6 (6.4) | 0.34 ± 1.38 | 0.38 ± 1.56 | |  | 0 (0.0) | 0.00 ± 0.00 | 0.00 ± 0.00 | |
| *Cotrimoxazole* |  | 2 (2.1) | 0.06 ± 0.41 | 0.07 ± 0.46 | |  | 0 (0.0) | 0.00 ± 0.00 | 0.00 ± 0.00 | |
| *Clarithromycin* |  | 1 (1.1) | 0.10 ± 0.98 | 0.11 ± 1.11 | |  | 0 (0.0) | 0.00 ± 0.00 | 0.00 ± 0.00 | |
| *Ear drops* |  |  |  |  | |  |  |  |  | |
| *Otalgan®* |  | 5 (5.3) | 0.16 ± 1.52 | 0.18 ± 1.72 | |  | 5 (3.9) | 0.12 ± 1.31 | 0.14 ± 1.48 | |
| *Sofradex®* |  | 1 (1.1) | 0.58 ± 2.48 | 0.65 ± 2.80 | |  | 1 (0.8) | 0.43 ± 2.16 | 0.49 ± 2.44 | |
| *Ofloxacin* |  | 1 (1.1) | 0.22 ± 2.18 | 0.25 ± 2.46 | |  | 0 (0.0) | 0.17 ± 1.89 | 0.19 ± 2.13 | |
| *Bacicoline B drops* |  | 1 (1.1) | 0.01 ± 0.05 | 0.01 0.06 | |  | 1 (0.8) | 0.00 ± 0.00 | 0.00 ± 0.00 | |
| **Total healthcare costs** |  |  | **77.74 ± 62.59** | **87.75 ± 70.65** | |  |  | **59.24 ± 33.86** | **66.87 ± 38.22** | |
| **Patient and family costs** |  |  |  |  | |  |  |  |  | |
| Travel expenses |  |  |  |  | |  |  |  |  | |
| *Fuel costs* |  | 7 (7.4) | 0.13 ± 0.52 | 0.15 ± 0.59 | |  | 1 (0.8) | 0.06 ± 0.60 | 0.07 ± 0.68 | |
| *Parking costs* |  | 7 (7.4) | 0.29 ± 1.20 | 0.33 ± 1.35 | |  | 1 (0.8) | 0.14 ± 1.38 | 0.16 ± 1.56 | |
| Over-the-counter medication |  |  |  |  | |  |  |  |  | |
| *Paracetamol* | *** | 83 (88.3) | 3.15 ± 3.07 | 3.56 ± 3.47 | | *** | 106 (83.5) | 2.07 ± 3.00 | 2.34 ± 3.39 | |
| *Ibuprofen* | *** | 51 (54.3) | 0.93 ± 1.36 | 1.05 ± 1.54 | | *** | 27 (21.3) | 0.39 ± 1.23 | 0.44 ± 1.39 | |
| *Xylometazoline nasal spray* | *** | 17 (18.1) | 1.08 ± 2.77 | 1.22 ± 3.13 | | *** | 12 (9.4) | 0.72 ± 2.81 | 0.81 ± 3.17 | |
| *Otrivin® nasal spray* | *** | 6 (6.4) | 0.29 ± 1.13 | 0.33 ± 1.28 | | *** | 11 (8.7) | 0.40 ± 1.31 | 0.45 ± 1.48 | |
| *Sodium chloride nasal spray* | *** | 19 (20.2) | 0.63 ± 1.29 | 0.71 ± 1.46 | | *** | 24 (18.9) | 0.60 ± 1.32 | 0.68 ± 1.49 | |
| *Complementary medicine* | *** | 17 (18.1) | 3.35 ± 9.08 | 3.78 ± 10.25 | | *** | 16 (12.6) | 3.46 ± 14.09 | 3.91 ± 15.90 | |
| *Cough syrup* | *** | 3 (3.2) | 0.77 ± 5.00 | 0.87 ± 5.64 | | *** | 6 (4.7) | 1.55 ± 9.63 | 1.75 ± 10.87 | |
| Childcare costs | *** | 12 (12.8) | 1.47 ± 8.70 | 1.66 ± 9.82 | | *** | 16 (12.6) | 4.48 ± 23.12 | 5.06 ± 26.10 | |
| **Total patient and family costs** | | | **12.08 ± 14.45** | **13.63 ± 16.31** | |  |  | **13.70 ± 28.48** | **15.46 ± 32.15** | |
| **Productivity losses** |  |  |  |  | |  |  |  |  | |
| Father |  | 33 (35.1) |  |  | |  | 45 (35.4) |  |  | |
| *Absenteeism* | *** |  | 86.09 ± 318.38 | 97.17 ± 359.36 | | *** |  | 102.18 ± 329.93 | 115.33 ± 372.40 | |
| *Presenteeism* | *** |  | 35.31 ± 162.25 | 39.86 ± 183.13 | | *** |  | 38.65 ± 168.75 | 43.63 ± 190.47 | |
| *Unpaid work* | *** |  | 43.22 ± 182.21 | 48.78 ± 205.66 | | *** |  | 28.73 ± 140.48 | 32.43 ± 158.56 | |
| *Total* | *** |  | 164.61 ± 526.62 | 185.80 ± 594.41 | | *** |  | 169.56 ± 457.83 | 191.39 ± 516.76 | |
| Mother |  | 53 (56.4) |  |  | |  | 71 (55.9) |  |  | |
| *Absenteeism* | *** |  | 126.51 ± 445.59 | 142.79 ± 502.95 | | *** |  | 138.50 ± 598.28 | 156.33 ± 675.29 | |
| *Presenteeism* | *** |  | 55.71 ± 199.27 | 62.88 ± 224.92 | | *** |  | 46.68 ± 178.16 | 52.69 ± 201.09 | |
| *Unpaid work* | *** |  | 76.46 ± 172.46 | 86.30 ± 194.66 | | *** |  | 106.87 ± 284.82 | 120.63 ± 321.48 | |
| *Total* | *** |  | 261.67 ± 573.10 | 295.35 ± 646.87 | | *** |  | 292.04 ± 748.10 | 329.63 ± 844.40 | |
| **Total productivity losses ^‡^** |  |  | **426.28 ± 947.15** | **481.15 ± 1069.07** | |  |  | **461.60 ± 916.83** | **521.02 ± 1034.84** | |
| **Total** |  |  |  |  | |  |  |  |  | |
| **Total healthcare costs** |  |  | **77.74 ± 62.59** | **87.75 ± 70.65** | |  |  | **59.24 ± 33.86** | **66.87 ± 38.22** | |
| **Total patient costs** |  |  | **12.08 ± 14.45** | **13.63 ± 16.31** | |  |  | **13.70 ± 28.48** | **15.46 ± 32.15** | |
| **Total productivity losses** |  |  | **426.28 ± 947.15** | **481.15 ± 1069.07** | |  |  | **461.60 ± 916.83** | **521.02 ± 1034.84** | |
| **Total costs** |  |  | **516.10 ± 949.69** | **582.53 ± 1071.93** | |  |  | **534.55 ± 920.55** | **603.36 ± 1039.04** | |

Two outliers in the control group have been removed; one with five-day admission for mastoiditis, one with unrealistic productivity losses.

ED: emergency department; GP: general practitioner, N/A: not applicable, OPD: out-patient department, SD: standard deviation

**^‡^** Per child, calculated as productivity losses of mother and/or father combined.

*missing values were imputed
